# Call for Breast Cancer Risk Factor Education in Countries With Limited Health Care Resources

**DOI:** 10.1200/JGO.2016.007781

**Published:** 2017-01-11

**Authors:** Tara J. Rick, Judith J. Merinyo

**Affiliations:** **Tara J. Rick**, St Catherine University, St Paul, MN; **Judith J. Merinyo**, Arusha Lutheran Medical Centre, Arusha, Tanzania

## TO THE EDITOR:

The recent article by Kennedy et al^1^ highlights the importance of opportunistic breast cancer education and increasing use of screening services in rural Honduras. Breast education programs that lead to increased awareness have an impact on reducing breast cancer–related mortality and is an inexpensive and important strategy,^2^ regardless of the availability of mammography and adjuvant therapy. We believe that in addition to education about self- and clinical breast examination, emphasis should be focused on the importance of reducing modifiable risk factors and, more importantly, the education of health care providers. This is particularly important as low- and middle-income countries increasingly adopt a Western lifestyle, which increases the risk of breast cancer.^3^ In addition, community-level health care providers should be a reliable source of breast health education in any setting.

As in Latin America, breast cancer is the most prevalent cancer in African women, and like Honduras, Tanzania has limited access to cancer education, screening, and treatment.^4^ To better understand breast cancer awareness and knowledge of risk factors in the general population, we surveyed 98 individuals in Arusha, Tanzania. The participants were patients, visitors, and nurses present in a general outpatient waiting room at a private general hospital. The survey was written in both English and Swahili (the local language) and asked dichotomous questions about whether patients were aware of breast cancer and whether they believed that it could be treated if diagnosed early. Breast cancer risk factor knowledge was assessed by a multiple-choice question that listed both true risk factors and known common regional misconceptions. Most of the participants were female (62.2%), the median age was 35 years (interquartile range, 28 to 48), 69% had a secondary education or higher, and 85.7% reported being employed or a student (Table 1).

**Table 1:**
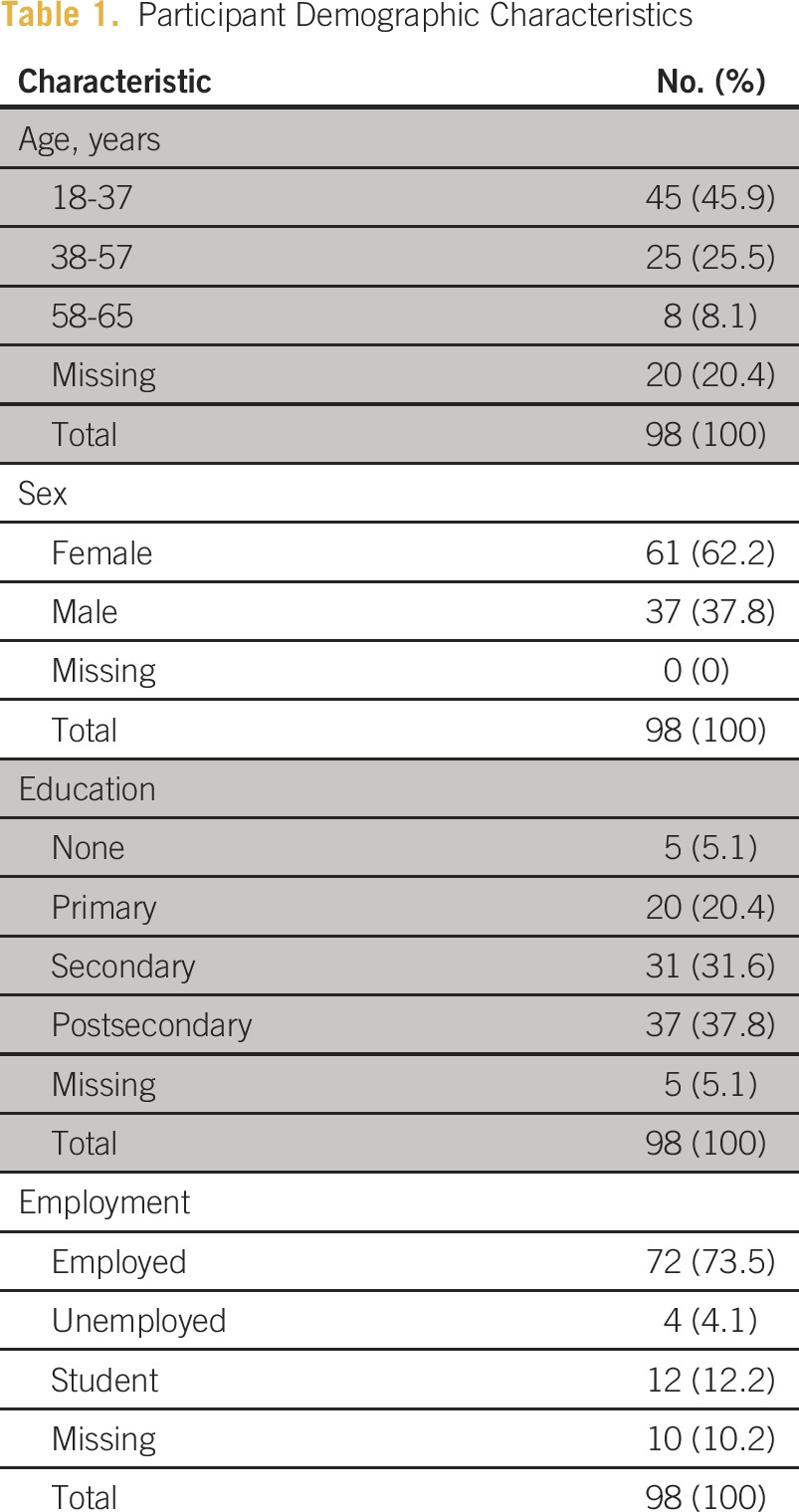
Participant Demographic Characteristics

Almost all surveyed had heard of breast cancer (96%), and most were aware it could be treated if detected early (93%). However, in the multiple-choice question, a notable 65% chose keeping money under the brassiere as a risk factor for breast cancer, including 77% of nine nurses surveyed (Table 2). Fifty percent of participants correctly identified family history as a risk factor, followed by alcohol consumption (38%), diet (38%), and increasing age (27%). Of note, several participants also believed that scratching the breast (20%), wearing a brassiere (19%), and being bitten by a child during breast-feeding (17%) are risk factors.

**Table 2:**
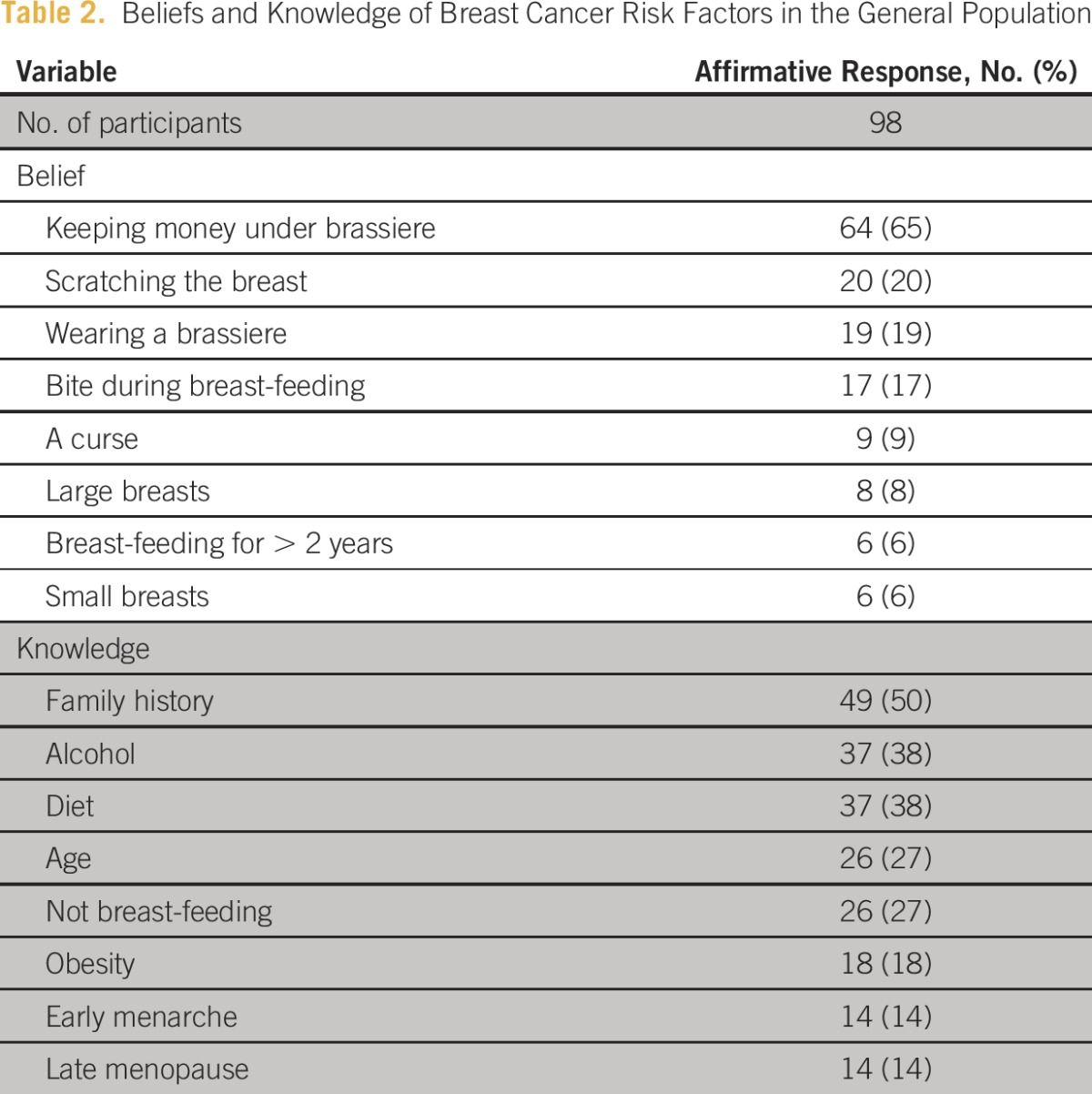
Beliefs and Knowledge of Breast Cancer Risk Factors in the General Population

On the basis of the survey findings, we extended the risk factor questions to 44 medical staff members in the community, including physicians, clinical officers, assistant medical officers, and nurses. We found that 76% identified keeping money under the brassiere as a risk factor for breast cancer, which was consistent with the belief of the general population. We then provided an educational session to health care providers with a focus on risk factors and early signs of cancer.

Similarly to Kennedy et al,^1^ our study highlights the emergent need for breast cancer education of the general population in countries with limited health care resources. The study also emphasizes the need for cancer-related risk factor education for community-level health care providers because providers are key in educating patients about prevention and early detection of breast cancer, especially in areas with limited screening and adjuvant treatment capabilities.
